# Supplementary material for: Stem-cell-ubiquitous genes spatiotemporally coordinate division through regulation of stem-cell-specific gene networks
Source: Nat Commun. 2019 Dec 6;10:5574. doi: 10.1038/s41467-019-13132-2 (PMC6897965; doi:10.1038/s41467-019-13132-2)
Supplement: Supplementary file 2 — Description of Additional Supplementary Files [file 41467_2019_13132_MOESM2_ESM.pdf]

Description of Additional Supplementary Files

Supplementary Data 1. Differentially enriched genes in stem cells. Differential enrichment of the 9266 stem cell-enriched genes across the different stem cell types. Enrichment is defined as fold change > 2 and q-value < 0.06 from PoissonSeq versus all other stem cells. Each gene is enriched in the stem cells with a 1 in the corresponding column. The last column is the total number of cells in which each gene is enriched. Cell-ubiquitous genes (red) are defined as genes enriched in 4 or more stem cells, while cell-specific genes (blue) are defined as genes enriched in 3 or less stem cells.

Supplementary Data 2. Normalized motif scores. The NMS score and rank are shown for each gene in the network. Only genes with an NMS score > 0 are ranked. Green genes have a known stem cell function and were used to validate the NMS score. The NMS score of TCX2 is highlighted in yellow.

Supplementary Data 3. Biological validation of TCX2 regulators and targets. The NMS rank and percentile for each of the 48 genes in the TCX2 first-neighbor TF network are provided. Genes are classified as cell-specific if they are enriched in 3 or less stem cell types. Genes of interest have NMS scores > 0. Mutant obtained: the mutant was obtained and validated in this paper. Phenotype: the obtained mutant did or did not show a stem cell phenotype. Relation to TCX2: the position of the gene in the network.

Supplementary Data 4. Validation of predicted TCX2 direct targets. Edges are listed as well as the cell they were predicted in, if they were validated by DAPSeq, if they were validated by RNASeq, if the predicted cell matched at least one of the DE cells, and if the predicted sign matched the known DE sign.

Supplementary Data 5. Genes differentially expressed in the *tcx2* mutant stem cell types and root tip. Each tab in the file represents a different pairwise comparison. For the cell type specific data, differential expression was defined as q<0.05 and fold change >2 based on the cutoff for the stem cell transcriptional profile. For the root tip data, differential expression was defined as q<0.5 and fold change >1.5 based on the expression of TCX2 in its own mutant. The 175 genes used in the TCX2 GRN that changes over time are denoted.

Supplementary Data 6. Sensitivity analysis. Results for the sensitivity analysis performed on each set of equations (4D to 4D 8H; 4D 8H to 4D 16H; 4D 16H to 5D) are shown. The total Sobol index was averaged across all variables for each of 10 technical replicates. Sensitive parameters are highlighted and have a p-value < 0.05 compared to the denoted control parameter. Yellow parameters were directly estimated from the stem cell time course or experimentally determined using scanning FCS. Green parameters were estimated using simulated annealing on the stem cell time course.

Supplementary Data 7. Parameter values. Note that all parameter values are constant across the entire model (4D to 5D). All initial conditions are the average values at 4D from the stem cell time course.

Supplementary Data 8. Model prediction of TCX2 expression. FC = fold change.

Supplementary Data 9. T-DNA lines used in this study.

Supplementary Data 10. Parameters used for GRN inference.

Supplementary Data 11. qPCR primers used in this study.
