# Supplementary material for: Stem-cell-ubiquitous genes spatiotemporally coordinate division through regulation of stem-cell-specific gene networks
Source: Nat Commun. 2019 Dec 6;10:5574. doi: 10.1038/s41467-019-13132-2 (PMC6897965; doi:10.1038/s41467-019-13132-2)
Supplement: Supplementary file 15 — Reporting Summary [file 41467_2019_13132_MOESM15_ESM.pdf]

nature research

Corresponding author(s): Sozzani, Rosangela

Last updated by author(s): Sep 19, 2019

Reporting Summary

Nature Research wishes to improve the reproducibility of the work that we publish. This form provides structure for consistency and transparency in reporting. For further information on Nature Research policies, see Authors & Referees and the Editorial Policy Checklist.

Statistics

For all statistical analyses, confirm that the following items are present in the figure legend, table legend, main text, or Methods section.

n/a	Confirmed	
☐	☒	The exact sample size (*n*) for each experimental group/condition, given as a discrete number and unit of measurement
☐	☒	A statement on whether measurements were taken from distinct samples or whether the same sample was measured repeatedly
☐	☒	The statistical test(s) used AND whether they are one- or two-sided<br>*Only common tests should be described solely by name; describe more complex techniques in the Methods section.*
☒	☐	A description of all covariates tested
☐	☒	A description of any assumptions or corrections, such as tests of normality and adjustment for multiple comparisons
☐	☒	A full description of the statistical parameters including central tendency (e.g. means) or other basic estimates (e.g. regression coefficient) AND variation (e.g. standard deviation) or associated estimates of uncertainty (e.g. confidence intervals)
☐	☒	For null hypothesis testing, the test statistic (e.g. *F*, *t*, *r*) with confidence intervals, effect sizes, degrees of freedom and *P* value noted<br>*Give P values as exact values whenever suitable.*
☒	☐	For Bayesian analysis, information on the choice of priors and Markov chain Monte Carlo settings
☒	☐	For hierarchical and complex designs, identification of the appropriate level for tests and full reporting of outcomes
☒	☐	Estimates of effect sizes (e.g. Cohen's *d*, Pearson's *r*), indicating how they were calculated

Our web collection on statistics for biologists contains articles on many of the points above.

Software and code

Policy information about availability of computer code

Data collection	ZEN software was used to collect confocal imaging data
Data analysis	Bowtie, Tuxedo, and RSubread were used to obtain FPKM values for all RNA-seq data<br>PoissonSeq was used for all differential expression analysis<br>InteractiveVenn (http://www.interactivenn.net/) was used for Venn diagram construction<br>Cytoscape (http://cytoscape.org/) was used for network visualization<br>RTP-STAR (http://github.com/nmclark2/RTP-STAR) was used for all network inference<br>BioVisionTracker (https://github.com/edbuckne/BioVision_Tracker) was used for YFP/GFP tracking<br>MATLAB code for the ODE model that support the findings of this study are available in figshare [https://doi.org/10.6084/m9.figshare.c.4539071.]

For manuscripts utilizing custom algorithms or software that are central to the research but not yet described in published literature, software must be made available to editors/reviewers. We strongly encourage code deposition in a community repository (e.g. GitHub). See the Nature Research guidelines for submitting code & software for further information.

Data

Policy information about availability of data

All manuscripts must include a data availability statement. This statement should provide the following information, where applicable:

- Accession codes, unique identifiers, or web links for publicly available datasets
- A list of figures that have associated raw data
- A description of any restrictions on data availability

Sequence data that support the findings of this study have been deposited in GEO with the primary accession codes #GSE98204[https://www.ncbi.nlm.nih.gov/geo/query/acc.cgi?acc=GSE98204], GSE123984 [https://www.ncbi.nlm.nih.gov/geo/query/acc.cgi?acc=GSE123984], and GSE131988 [https://www.ncbi.nlm.nih.gov/

geo/query/acc.cgi?acc=GSE131988]. The raw images and data used for GRN inference and biological validation are available in figshare [https://doi.org/10.6084/m9.figshare.c.4539071.] The source data underlying Figure 3 and Supplementary Figures 3, 4, 5, 8, and 9 are provided as a Source Data file.

Field-specific reporting

Please select the one below that is the best fit for your research. If you are not sure, read the appropriate sections before making your selection.

☒ Life sciences ☐ Behavioural & social sciences ☐ Ecological, evolutionary & environmental sciences

For a reference copy of the document with all sections, see nature.com/documents/nr-reporting-summary-flat.pdf

Life sciences study design

All studies must disclose on these points even when the disclosure is negative.

Sample size	No statistical methods were used to predetermine sample size. For high-throughput sequencing, at least 2 replicates were collected for each sample. For all other data (images, etc) at least 5 replicates were collected for each group.
Data exclusions	For the stem cell transcriptional profile and time course, some replicates were excluded from downstream analysis due to poor sequence quality. Some confocal images were excluded from analysis due to poor image quality or issues with sample preparation (i.e. damaged or stressed roots)
Replication	Multiple biological replicates were collected for all experiments to ensure that the results are replicable.
Randomization	This is not relevant for our study as seeds are collected/chosen randomly for all experiments.
Blinding	Investigators were not blinded to different comparisons during this study.

Reporting for specific materials, systems and methods

We require information from authors about some types of materials, experimental systems and methods used in many studies. Here, indicate whether each material, system or method listed is relevant to your study. If you are not sure if a list item applies to your research, read the appropriate section before selecting a response.

Materials & experimental systems

n/a	Involved in the study	
☒	☐	Antibodies
☒	☐	Eukaryotic cell lines
☒	☐	Palaeontology
☒	☐	Animals and other organisms
☒	☐	Human research participants
☒	☐	Clinical data

Methods

n/a	Involved in the study	
☒	☐	ChIP-seq
☐	☒	Flow cytometry
☒	☐	MRI-based neuroimaging

Flow Cytometry

Plots

Confirm that:

☒ The axis labels state the marker and fluorochrome used (e.g. CD4-FITC).

☒ The axis scales are clearly visible. Include numbers along axes only for bottom left plot of group (a 'group' is an analysis of identical markers).

☒ All plots are contour plots with outliers or pseudocolor plots.

☒ A numerical value for number of cells or percentage (with statistics) is provided.

Methodology

Sample preparation	Approximately 1-2mm of the root tip was cut and protoplasted using a solution containing cellulase and pectolyase.
Instrument	Dako Cytomation MoFlo
Software	Summit Software
Cell population abundance	Cell population abundance was determined based on the reported numbers of cells in each gate from the software. After RNA

Cell population abundance	was extracted we checked that the RNA concentration correlated with the number of cells collected to validate the predicted abundance.
Gating strategy	For the stem cell transcriptomic profile we used a combination of 3 gating strategies as shown in Supplementary Figure 10: 1) Side-scatter (SS) versus log-fluorescence (FL 1 Log). GFP-positive cells had log-fluorescence > 10^1.5. (2) Counts vs log fluorescence of cells in the region (1). GFP-positive cells had log-fluorescence > 10^2.5. 3) Pulse width vs forward scatter (FS) of GFP-positive cells to remove doublets. For the GFP-negative control we considered the GFP-negative cells to have log fluorescence < 10^1 and side scatter < 128. Pulse width vs forward scatter (FS) was used on the GFP-negative cells to remove doublets.

☒ Tick this box to confirm that a figure exemplifying the gating strategy is provided in the Supplementary Information.
